# Knowledge, attitude, and practice of medication and its influencing factors among residents in western China: a large-scale cross-sectional study

**DOI:** 10.3389/fmed.2024.1303672

**Published:** 2024-02-19

**Authors:** Hong-xia Shi, Xue Tan, Jian-qin Shu, Jun Zhou, Si-yuan Dan, Lin Yang, Ze-lian Chen

**Affiliations:** ^1^Department of Pharmacy, West China Hospital, Sichuan University, Chengdu, Sichuan Province, China; ^2^Department of Pharmacy, People’s Hospital of Karamay, Karamay Xinjiang, China

**Keywords:** knowledge attitude and practice (KAP), rational drug use, western China, questionnaire survey, influencing factor

## Abstract

**Background:**

This study aimed to understand the knowledge, attitude, and practice (KAP) of drug use among residents in western China and its influencing factors for accurately designing the knowledge, contents, and methods of popular science activities for safe drug use among residents to provide a reference for conducting rational drug use educational activities and improving residents’ level of safe drug use.

**Methods:**

A cross-sectional questionnaire survey was conducted to investigate the KAP of medication among western China residents and its influencing factors from March to April 2023. Each question option was assigned a score according to logic, and the risk factors for resident medication safety KAP were explored through univariate and logistic regression analyses.

**Results:**

A total of 7,557 valid questionnaires were collected, with an effective recovery rate of 96.7%. The average scores of KAP were 72.77 ± 22.91, 32.89 ± 10.64, and 71.27 ± 19.09, respectively. In the evaluation criteria of the questionnaire, the score of medication knowledge reached “good,” and the score of attitude and practice was “average.” Multiple linear regression analysis indicated that male sex and low education level were significant factors affecting the lack of drug knowledge among residents. Old age and low education level were the factors of poor attitude toward medication. The low condition of medical security was a factor in residents’ irregular drug use behavior.

**Conclusion:**

The overall level of rational drug use among residents in western China is good, but there are still some inconsistencies. Rational drug use education should be conducted according to the risk points of residents in drug safety KAP to further improve the level of rational drug use of residents.

## Introduction

With the development of society, economy, and culture and the transformation of medical service modes, people are paying increasing attention to medical health. In terms of chronic diseases and common diseases, most people choose self-medication. Through self-medication, people can prevent and relieve mild or chronic diseases promptly, reducing the economic cost of patients, shortening the time of medical treatment, and reducing the pressure on medical institutions (1). Because of differences in regions, culture, and economic levels, residents may have poor knowledge of rational drug use, poor understanding ability, poor compliance, and other problems, thus increasing the risk of drug use (2–4). Improper drug use may harm the body, delay the treatment of diseases, produce adverse drug reactions and drug-induced diseases, and lead to drug abuse and waste of medical resources (5). The knowledge, attitude, and practice (KAP) model, also known as the “knowledge and belief” model, is an approach to studying the influence of interviewees’ knowledge, attitude, and behavior on certain things. It divides the change in people’s behavior into three continuous processes: acquiring knowledge, generating belief, and forming behavior (6). There is a gap in KAP-related research in western China (7–10). To solve this problem, the authors performed a KAP investigation and research on medication risk among residents in this region to understand the current situation, influencing factors, and risk points of rational drug use among residents; formulate the content of popular science education on rational drug use for different groups; effectively improve the level and awareness of rational drug use among residents; reduce dangerous drug use behavior; and provide a scientific basis for intervention strategies related to rational drug use.

## Methods

### Subjects

A cross-sectional questionnaire survey was conducted among permanent residents in western China from March to April 2023. All participants provided written informed consent, including minors, who also obtained the consent of their guardians.

### Research methods

The questionnaire was created into an online questionnaire using the Questionnaire Star survey platform, and the online questionnaire was disseminated to residents in western China through WeChat.

### Result evaluation criteria

This study used the KAP model (11), and the KAP Questionnaire on Medication Risk of Chinese Residents designed by the Science and Technology Development Center of China Pharmaceutical Association was used as the template for questionnaire design. A five-level scale was used to quantify the degree of agreement, the degree of necessity, and the actual frequency of occurrence. The contents of the questionnaire included the following: (1) demographic characteristics of the respondents, including gender, age, monthly income, and education level, a total of eight questions; (2) knowledge of drugs and drug use, including intravenous medication is more effective than oral medicine and whether antibacterial drugs are anti-inflammatory drugs, a total of 33 questions (a score of <33 indicates better drug knowledge, 33–66 indicates excellent, 67–99 indicates good, 100–132 indicates passing, and ≥ 133 indicates failing); (3) attitude toward knowledge education of rational drug use, including whether they have participated in lectures on knowledge of rational drug use in the community and whether they have participated in community or street counseling services by pharmacists in the community, a total of 11 questions (the higher the score, the better the attitude; a score of 11–22 indicates failing, 23–33 indicates passing, 34–44 indicates good, and ≥ 45 indicates excellent); (4) real drug use behavior, including checking the expiration date before drug use and checking the expiration date regularly, a total of 30 questions (a higher score indicates better drug use behavior; 30–60 indicates failing, 61–90 indicates passing, 91–120 indicates good, and ≥ 121 indicates excellent).

### Questionnaire quality control

The system is automatically set thus that only one questionnaire can be filled out and submitted per each Internet protocol address so as to avoid the repeated submission of questionnaires by respondents. After the questionnaire was completed, the data were exported, and SPSS software was used to sort out the logical relationship between questions and answers; screen and filter multiple options, missing items, and all the same options; and automatically extract valid questionnaires to avoid the inclusion of invalid questionnaires.

### Statistical analysis

Data in this study were entered into Excel 2016 and analyzed using the SPSS 22.0 software package. General information was described in terms of frequency and component ratio, and measurements are expressed as $\bar{x}$± SD. Univariate analysis was performed by independent sample t test or variance analysis. A multiple linear regression model was used to calculate the regression coefficient and compare the influence of each causal variable on the result variable. The correlation between different scores was analyzed, and *p* < 0.05 was considered statistically significant.

## Results

### Basic information of surveyed residents

**Figure 1:**
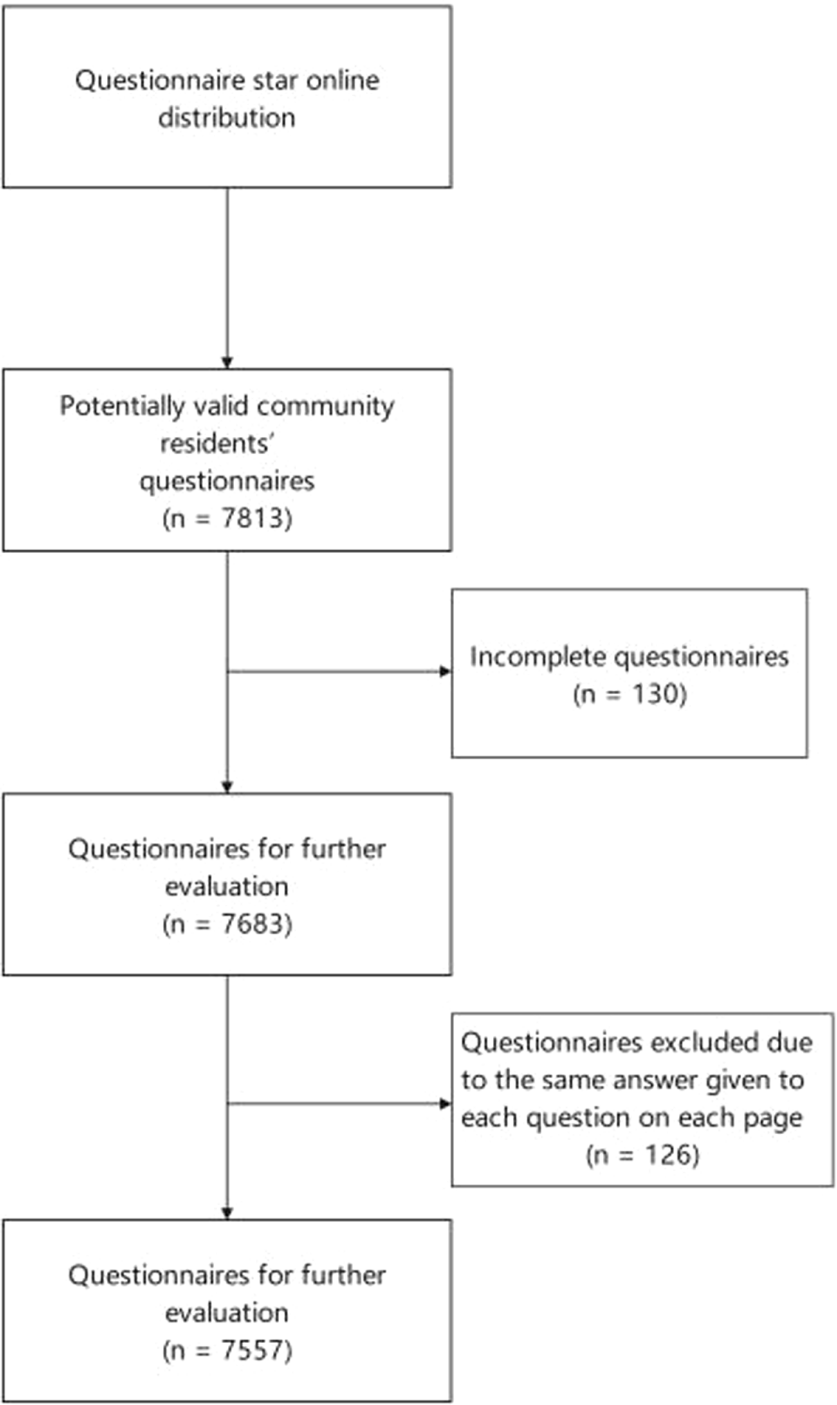
Flowchart of the selection of valid questionnaires.

A total of 7,813 questionnaires were distributed, 7,557 of which were valid, with an effective response rate of 96.7%. Figure 1 presents the details of the selection process. The univariate analysis of KAP scores of residents with different genders, ages, monthly income, residence, medical insurance status, education levels, working status, and occupations is presented in Table 1. In terms of the different occupations, respondents included 2,455 men (32.5%) and 5,102 women (67.5%), aged 35–49 years (79.4%). Their average monthly income ranged from 2,000 to 4,000 yuan (34.5%) and 4,000 to 6,000 yuan (32.2%). A total of 85.1% of respondents were currently employed, and 94.7% were urban residents. The education level was 23.8% in middle school and 23.7% in high school. Up to 81.0% of respondents were covered by social basic medical insurance.

**Table 1:** Demographic characteristics of residents and KAP scores of drug use risk.

Demographic features	Group	*n*	Percentage	Knowledge		Practice		Attitude	
				$\bar{x}$± SD	*p*	$\bar{x}$± SD	*p*	$\bar{x}$± SD	*p*
Gender	Male	2,455	32.5	72.99 ± 24.37	0.036^*^	70.64 ± 20.73	0.299	32.68 ± 11.06	0.201
	Female	5,102	67.5	71.85 ± 20.91		71.11 ± 16.90		33.01 ± 10.21	
Age		400	5.3	75.14 ± 29.81	0.002^**^	72.42 ± 25.39	0.057	32.47 ± 12.47	0.005^**^
	35–49 years	5,997	79.3	71.75 ± 21.43		70.67 ± 17.68		32.75 ± 10.34	
	50–64 years	1,102	14.6	73.51 ± 22.10		71.94 ± 17.72		33.93 ± 10.52	
	65 years or older	58	0.8	75.48 ± 25.28		72.10 ± 23.32		32.55 ± 10.48	
Monthly income	<1,000	417	5.5	73.52 ± 24.13	0.000^**^	71.70 ± 20.36	0.521	32.13 ± 11.20	0.211
	1,000–2,000	719	9.5	74.72 ± 23.72		71.41 ± 20.15		32.68 ± 10.74	
	2,000–4,000	2,606	34.5	73.10 ± 22.36		71.23 ± 18.12		33.23 ± 10.39	
	4,000–6,000	2,431	32.2	71.17 ± 20.97		70.55 ± 17.16		32.88 ± 10.40	
	>6,000	1,384	18.3	70.70 ± 21.86		70.70 ± 18.55		32.67 ± 10.50	
Place of residence	Urban areas	7,159	94.7	71.93 ± 22.02	0.000^**^	70.91 ± 18.14	0.285	32.96 ± 10.52	0.054
	Rural areas	398	5.3	77.34 ± 22.92		71.91 ± 19.80		31.91 ± 9.89	
Health insurance coverage	Basic social medical insurance	6,120	81	71.35 ± 21.61	0.000^**^	70.56 ± 17.80	0.000^**^	32.96 ± 10.48	0.22
	Publicly funded health care	527	7	74.41 ± 22.12		72.44 ± 18.17		33.21 ± 10.11	
	Out-of-pocket payment	478	6.3	75.92 ± 21.78		71.52 ± 19.09		31.82 ± 10.42	
	Commercial insurance	86	1.1	75.66 ± 22.21		72.00 ± 18.80		33.09 ± 10.06	
	Others	346	4.6	78.24 ± 28.47		74.64 ± 23.35		32.95 ± 11.42	
Education level	Primary school	501	6.6	75.99 ± 24.55	0.000^**^	71.87 ± 20.68	0.039^*^	33.08 ± 11.54	0.109
	Middle school	1,799	23.8	74.98 ± 23.39		71.88 ± 19.05		33.48 ± 10.79	
	High school/technical secondary school	1,793	23.7	72.43 ± 22.60		70.37 ± 19.25		32.90 ± 10.76	
	High school/technical secondary school	1,716	22.7	71.21 ± 20.54		71.16 ± 16.91		32.62 ± 10.15	
	Bachelor’s degree	1,661	22	69.00 ± 19.84		70.09 ± 16.19		32.51 ± 9.83	
	Postgraduate student	87	1.2	69.98 ± 28.92		71.20 ± 24.63		32.90 ± 11.02	
Employment	Retired	361	4.8	73.95 ± 25.18	0.013^*^	71.95 ± 19.12	0.114	33.83 ± 11.23	0.074
	Unemployed/jobless	766	10.1	74.02 ± 22.18		72.03 ± 18.35		32.31 ± 10.40	
	Currently employed	6,430	85.1	71.90 ± 21.89		70.78 ± 18.16		32.92 ± 10.46	
Occupation	Medical institutions	248	3.3	65.23 ± 23.68	0.000^**^	68.86 ± 19.87	0.418	34.90 ± 12.39	0.018^*^
	Government cadres	1,217	16.1	72.52 ± 22.29		71.07 ± 18.85		32.69 ± 10.61	
	Others	1,168	15.5	73.26 ± 21.19		71.15 ± 17.75		32.72 ± 10.49	
	Enterprise workers	1,607	21.3	72.81 ± 21.30		70.83 ± 17.68		32.51 ± 10.09	
	Teachers	294	3.9	68.22 ± 19.77		70.72 ± 16.68		33.05 ± 9.58	
	Government cadres	365	4.8	70.46 ± 22.86		71.01 ± 17.15		33.36 ± 10.43	
	Business managers	441	5.8	68.85 ± 20.68		69.67 ± 18.02		32.19 ± 10.77	
	Others	2,217	29.3	73.34 ± 23.03		71.40 ± 18.74		33.22 ± 10.54	
Total				72.77 ± 22.91		71.27 ± 19.09		32.89 ± 10.64	

KAP, knowledge, attitude, and practice. ^*^ *p* < 0.05; ^**^ *p* < 0.01.

### Scores of residents’ medication knowledge in western China

The average score of medication knowledge of residents in western China was 70.47 ± 22.29, and the overall score of medication knowledge was good according to the scoring standard. A total of 2,810 respondents (37.18%) were rated as excellent, 4,375 respondents (57.89%) were rated as good, 295 respondents (3.90%) were rated as passing, and 78 respondents (1.03%) were rated as failing. There were statistically significant differences in medication knowledge scores among respondents with different genders, ages, monthly income, residence, medical security status, education levels, working status, and occupations (*p* < 0.05).

### Scores of drug use behavior among residents in western China

The average score of drug use behavior of residents in western China was 68.64 ± 19.10 points, and the overall knowledge score was average according to the scoring standard. A total of 58 respondents (0.77%) were rated as excellent, 348 (4.61%) were rated as good, 5,464 (72.30%) were rated as passing, and 1,687 (22.32%) were rated as failing. There were statistically significant differences in medication behavior scores among respondents with different medical insurance status (*p* < 0.05).

### Scores of residents’ attitude toward medication in western China

The average score of residents’ attitude toward medication in western China was 30.77 ± 10.60 points, and the overall attitude score was average according to the scoring standard. A total of 827 respondents (10.94%) were rated as excellent, 1,877 (24.84%) were rated as good, 3,628 (48.01%) were rated as passing, and 1,225 (1.6.21%) were rated as failing. There were statistically significant differences in medication knowledge scores among respondents of different ages, working conditions, and occupations (*p* < 0.05).

### Influencing factors of KAP drug use among residents in western China

The scores of knowledge, behavior, and attitude were assigned as dependent variables. Demographic characteristics were independent variables (Table 2). Tables 3–5 present the results of multiple linear regression analysis.

**Table 2:** Assignment of dependent and independent variables.

Variables	Value assignment
Gender	Male = 1 and female = 2
Age	19–34 years = 1, 35–49 years = 2, 50–64 years = 3, and 65 years or older = 4
Monthly income	<1,000 CNY = 1, 1,001–2,000 CNY = 2, 2,001–4,000 CNY = 3, 4,001–6,000 CNY = 4, and ≥ 6,001 CNY = 5
Place of residence	Urban = 1 and rural = 2
Medical insurance coverage	Social basic medical insurance = 1, publicly funded health care = 2, out-of-pocket payment = 3, commercial insurance = 4, and others = 5
Education level	Primary school = 1, middle school = 2, high school/technical secondary school = 3, junior college = 4, bachelor’s degree = 5, and graduate student = 6
Employment	Retired = 1, unemployed/jobless = 2, and currently employed = 3
Occupation	Medical institutions = 1, company employees = 2, freelancers = 3, enterprise workers = 4, teachers = 5, government cadres = 6, business managers = 7, and others = 8

**Table 3:** Factors influencing the medication knowledge of residents in the central and western regions.

Variables	Nonstandardized coefficient		Standardization coefficient	*t*	*p*	VIF
	*B*	Standard error	Beta			
*C*	76.312	2.994	–	25.488	0.000^**^	–
Gender	−1.221	0.556	−0.026	−2.194	0.028^*^	1.068
Age	0.49	0.56	0.01	0.876	0.381	1.073
Monthly income	−0.352	0.276	−0.017	−1.274	0.203	1.363
Place of residence	2.661	1.162	0.027	2.29	0.022^*^	1.059
Medical insurance coverage	1.39	0.262	0.063	5.301	0.000^**^	1.067
Education level	−1.396	0.225	−0.08	−6.202	0.000^**^	1.278
Employment	−0.512	0.535	−0.012	−0.956	0.339	1.143
Occupation	−0.11	0.11	−0.012	−1.004	0.316	1.08
*R*^2^	0.017					
F	*F* (8, 7,548)16.083, *p* = 0.000					
D-W	2.011					

^*^ *p* < 0.05; ^**^ *p* < 0.01.

**Table 4:** Factors influencing the drug use behavior of residents in central and western China.

Variables	Nonstandardized coefficient		Nonstandardized coefficient	*t*	*p*	VIF
	B	Standard error	Beta			
*C*	70.049	2.487	–	28.168	0.000^**^	–
Gender	0.492	0.462	0.013	1.065	0.287	1.068
Age	0.443	0.465	0.011	0.953	0.341	1.073
Monthly income	−0.006	0.229	0	−0.028	0.978	1.363
Place of residence	0.171	0.965	0.002	0.177	0.859	1.059
Medical insurance coverage	0.816	0.218	0.044	3.746	0.000^**^	1.067
Education level	−0.27	0.187	−0.019	−1.444	0.149	1.278
Employment	−0.433	0.445	−0.012	−0.974	0.33	1.143
Occupation	−0.01	0.091	−0.001	−0.107	0.915	1.08
*R*^2^	0.003					
*F*	*F* (8, 7,548)3.086, *p* = 0.002					
D-W	2.014					

^*^ *p* < 0.05; ^**^ *p* < 0.01.

**Table 5:** Factors affecting residents’ attitude toward medication in central and western China.

Variables	Nonstandardized coefficient		Nonstandardized coefficient	*t*	*p*	VIF
	B	Standard error	Beta			
*C*	32.374	1.431	–	22.619	0.000^**^	–
Gender	0.44	0.266	0.02	1.653	0.098	1.068
Age	0.792	0.268	0.035	2.959	0.003^**^	1.073
Monthly income	0.149	0.132	0.015	1.13	0.258	1.363
Place of residence	−1.249	0.556	−0.027	−2.248	0.025^*^	1.059
Medical insurance coverage	−0.112	0.125	−0.011	−0.892	0.372	1.067
Education level	−0.358	0.108	−0.043	−3.327	0.001^**^	1.278
Employment	0.057	0.256	0.003	0.223	0.824	1.143
Occupation	0.022	0.052	0.005	0.421	0.674	1.08
*R*^2^	0.004					
*F*	*F* (8, 7,548)3.325, *p* = 0.001					
D-W	1.995					

^*^ *p* < 0.05; ^**^ *p* < 0.01.

### Analysis of influencing factors of medication knowledge of residents in the central and western regions

The factors affecting medication knowledge were gender, residence, medical security status, education level, and other variables, which had no statistical significance (*p* > 0.05). Gender and education level were negatively correlated with medication knowledge, indicating that the medication knowledge of women was higher than that of men. The higher the education level, the lower the score of medication knowledge. Place of residence and medical security were positively correlated with medication knowledge. Better medical security and living in the city correspond to better medication knowledge.

### Analysis of influencing factors of drug use behavior of residents in western China

The factor influencing drug use behavior was medical security status, and there was no significant difference in other variables (*p* > 0.05). There was a positive correlation between medical security status and medication behavior, indicating that the better the medical security status, the more standardized the medication behavior.

### Analysis of influencing factors of drug use attitude of residents in western China

Age, place of residence, and education level were the factors that affected attitude toward medication, and the other variables had no statistical significance (*p* > 0.05). Age was positively correlated with attitude toward medication, indicating that the older the age, the more positive the attitude toward medication. There was a negative correlation between residence and education level, and attitude toward medication, indicating that residents in rural areas had a poor attitude toward medication. The higher the education level, the lower the attitude toward medication score.

## Discussion

### Risk analysis of residents’ knowledge of drug use in western China

The survey results indicated that the overall score of residents in western China was good, indicating that most residents have a high awareness of rational drug use. However, some residents still lack medication knowledge. A total of 20.04% of respondents believed that they should do their best to obtain intravenous medication and hang water when they are sick; A total of 22.66% of respondents believed that intravenous medication is more effective than oral medicine; 20.93% of respondents believed that drugs could be stopped or drug dosage could be reduced when their symptoms were alleviated, suggesting that it is still necessary to further strengthen the knowledge of rational drug use among the public. In addition, some residents lacked knowledge about antimicrobial drugs and their resistance. Misuse and abuse of antimicrobial drugs may lead to the emergence of drug-resistant bacteria (12–14). Bacterial resistance is among the top 10 global public health issues threatening mankind and has become a global concern (15). Some interviewees lacked knowledge of antibacterial drugs, and 55.99% of respondents believed that as long as they do not abuse antibiotics, there would be no resistance; 28.75% of respondents did not know whether antibacterial drugs were anti-inflammatory drugs; 34.44% of respondents were not certain whether antibiotics could be used for bacterial infections. Therefore, it is suggested that relevant departments jointly establish a publicity platform for rational drug use with medical institutions, communities, colleges and universities, and media to conduct regular and multilevel education and publicity for residents (16), increase residents’ awareness of rational drug use, and reduce the misuse and abuse of antibacterial drugs.

### Risk analysis of resident drug use in western China

Before purchasing and taking a drug, information related to the drug should be carefully read, such as drug usage, dosage, precautions, adverse reactions, and expiration date. The results of this survey indicated that 27.35% of respondents bought drugs by themselves on the basis of their experience or advertising. Only 26.8% of respondents purchased drugs with a doctor’s prescription. A total of 44.57% of respondents heeded the advice of salespeople when buying drugs in pharmacies. According to the Regulations for Implementation of the Drug Administration Law, drug retail enterprises dealing with prescription drugs and Class A nonprescription drugs shall have licensed pharmacists or other certified pharmaceutical technicians. By the end of March 2023 (17), there were a total of 725,998 licensed pharmacists within the registration period in China, with a month-over-month increase of 9,791, and there were 5.1 licensed pharmacists per 10,000 population. There were 659,998 licensed pharmacists registered in drug retail enterprises, accounting for 90.9% of the total number of registered pharmacists. Because of regional reasons, the uneven distribution of licensed pharmacists leads to a large gap in social pharmacies, and there are differences in the professional level of licensed pharmacists. Therefore, there are certain risks in guiding residents to rational drug use. Therefore, relevant departments should regularly organize professional and standardized training for licensed pharmacists and constantly improve the level and professional quality of licensed pharmacists.

In addition, 26.28% of respondents never or occasionally conducted regular checks on drugs stored at home; 32.83% of respondents never or occasionally observed the expiration date of drugs before taking them; 36.35% of respondents never or occasionally checked the storage conditions in the drug instructions before taking drugs; 45.54% of respondents dispose expired drugs at home. Most residents do not understand the concept of drug recycling and do not know the harm caused by expired drugs (18). Therefore, it is suggested that the relevant departments establish a recycling system for expired drugs (19, 20).

### Risk analysis of residents’ attitude toward drug use in western China

The survey results indicated that the frequency of residents participating in rational drug use knowledge seminars/educational activities was low. A total of 57.64% of respondents never or occasionally attended lectures on rational drug use in their place of residence; 53.77, 51.81, and 46.74% of respondents never or occasionally read the publicity materials on rational drug use issued by community/hospital/network, WeChat, and other services; 57.08% of respondents never or occasionally attend pharmacist community or street counseling services. Therefore, it is suggested that relevant departments should increase the frequency of rational drug use knowledge lectures/rational drug use educational activities and conduct scientific, standardized, and personalized rational drug use education for different populations (21). We need to continue to improve the residents’ level and awareness of the rational use of drugs.

In addition, 57.15 and 58.5% respondents considered it necessary or extremely necessary to conduct community/hospital rational drug use knowledge lectures; 59.37 and 60.01% of respondents considered it necessary or extremely necessary for community/hospital to issue publicity materials on rational drug use; 58.84% respondents believed that it is necessary or extremely necessary to disseminate publicity materials on rational drug use knowledge through the Internet and WeChat, indicating that residents are responsive to lecture/education on rational drug use knowledge. Therefore, it is suggested that relevant departments cooperate with medical institutions, communities, colleges and universities, media, and other services to establish a publicity platform for rational drug use, conduct community lectures, set up rational drug use consultation services in permanent places, popularize knowledge of rational drug use, and constantly improve residents’ awareness of rational drug use (22–24).

### Analysis of resident medication KAP and various factors

Multiple linear regression analysis indicated that the factors affecting drug use knowledge were gender, residence, medical security status, and education level. Most women are more mindful of rational drug use at home, and their knowledge of rational drug use was higher than that of men. Residents in rural areas with poor medical security and lower education levels have lower knowledge of rational drug use, and the situation is dismal. This may be related to the relatively backward education in the local area and the insufficient popularization of knowledge related to health education. Therefore, it is suggested that relevant departments attach importance to education, focusing on rural residents and residents with poor medical conditions, and strengthen the popularization of medical knowledge to improve the rational drug use level of residents.

The only factor affecting drug use behavior was medical security status, indicating that the better the medical security status, the more standardized the rational drug use behavior of residents. The factors influencing the attitude toward medication were age, place of residence, and education level, indicating that the residents’ attitude toward medication was more positive with increasing age, which may be because elderly people use more drugs in daily life and pay more attention to the knowledge of rational drug use. The poorer attitude of rural residents toward rational drug use may be related to the low frequency of lectures/educational activities on rational drug use in rural residents. Therefore, it is suggested that relevant departments conduct popular science education for different groups; for example, young people often use news media. Knowledge of rational drug use can be spread through social platforms such as Weibo and WeChat and short video platforms such as Douyin and Kuaishou. Elderly individuals often suffer from chronic diseases or multiple underlying diseases and may take multiple drugs at the same time. However, they have low awareness and knowledge levels of rational drug use and poor understanding ability (25) and urgently require drug use knowledge. Therefore, it is suggested that relevant departments regularly organize pharmacists to conduct public lectures and seminars on rational drug use in rural areas and communities, distribute popular science books, provide medication consultation and guidance, follow up medication, and establish a chronic management center throughout the process, which can reduce the amount of multidrug use in elderly patients, reduce the incidence of multidrug use, enhance behavioral self-management, and increase knowledge and belief in rational drug use, eventually improving safety and medication adherence behavior (26, 27). At the same time, these will constantly improve the overall quality of the science popularization team, establish a systematic and standardized science popularization system, provide a practical guarantee for promoting the widespread popularization of pharmacy science, promote the improvement of the scientific quality of safe drug use of residents, and actively help the implementation of the strategy of “Healthy China.”

## Conclusion

In summary, this survey objectively and accurately reflects the medication situation of residents in the western region of China. It was found that there are some common medication misconceptions and errors in rural areas with poor medical conditions and older residents. This indicates that to improve the rational medication level of residents and assist in the implementation of the Healthy China strategy, medical institutions should collaborate with communities, universities, media, and other services to establish a platform for promoting rational drug use and provide regular and multilevel education and publicity to residents. It is urgent to conduct rational drug use education.

## Data availability statement

The original contributions presented in the study are included in the article/supplementary material, further inquiries can be directed to the corresponding author.

## Ethics statement

The studies involving human participants were reviewed and approved by the Biomedical Ethics Committee of West China Hospital of Sichuan University. Written informed consent for participation in this study was provided by the participants’ legal guardians/next of kin.

## Author contributions

H-xS: Conceptualization, Methodology, Writing – original draft. XT: Data curation, Writing – review & editing. J-qS: Data curation, Formal analysis, Writing – review & editing. JZ: Methodology, Writing – review & editing. S-yD: Methodology, Writing – review & editing. LY: Conceptualization, Methodology, Writing – review & editing. Z-lC: Paper design, Review & supervision.
